# Pathways for Ukraine’s post-war nature recovery: Focus on forest socio-ecological systems

**DOI:** 10.1007/s13280-025-02263-0

**Published:** 2025-10-23

**Authors:** Mariana Melnykovych, Maria Nijnik, Oleksandr Soshenskyi, Sergiy Zibtsev, Ganna Lobchenko, Simo Sarkki, Natalia Voloshyna, Ihor Soloviy, Pavlo Kravets, Yevhenii Khan, Roman Yaroshchuk, William S. Keeton, Christian Rosset, Bernhard Pauli, Claude A. Garcia, Patrick O. Waeber

**Affiliations:** 1 School of Agriculture, Forest and Food Sciences HAFL, Bern University of Applied Sciences BFH, Länggasse85, 3052 Zollikofen, Switzerland; 2 The James Hutton Institute, Craigiebuckler, Aberdeen, AB15 8QH UK; 3 National University of Life and Environmental Sciences of Ukraine, 15 Heroiv Oborony St., Kyiv, 03041 Ukraine; 4 Regional Eastern Europe Fire Monitoring Center, 8 Buchmy St., Office 250, Kyiv, 02152 Ukraine; 5 WWF-Ukraine, Kyiv, Ukraine; 6 Cultural Anthropology, University of Oulu, PO Box 1000, 90014 Oulu, Finland; 7 Max Weber Institute for Advanced Cultural and Social Studies, Erfurt University, Nordhäuser Str. 63, 99089 Erfurt, Germany; 8 NGO FORZA, Agency for Sustainable Development of Carpathian Region, Mynayska 27/39, Uzhhorod, 88000 Ukraine; 9 Ukrainian National Forestry University, Gen. Chuprynky Str., 103, Lviv, 79057 Ukraine; 10 Ukrainian Research Institute of Forestry and Forest Melioration Named After G.M. Vysotsky, 86 Skovorody St., Kharkiv, 61024 Ukraine; 11 FSC Ukraine, Vasylkivska Str. 14, Kyiv, 03041 Ukraine; 12 Sumy National Agrarian University, 160 Herasyma Kondratieva Street, Sumy, 40000 Ukraine; 13 Rubenstein School of Environment and Natural Resources, and Gund Institute for Environment, University of Vermont, Burlington, VT 05401 USA; 14 Swiss Federal Institute of Technology ETH, 8092 Zurich, Switzerland; 15 Independent Researcher, Sarnen, Switzerland

**Keywords:** Anticipatory governance, “Build back better”, Climate change, Resilience, Socio-ecological innovation, War and military conflicts

## Abstract

Natural ecosystems face significant pressure from climate change, biodiversity loss, and increasing socio-economic and political stressors—all of which are exacerbated by military conflict. This paper focuses on Ukraine and examines the root causes of disturbances in the country’s forest socio-ecological systems (SES), as well as the impacts of the ongoing war, to identify recovery pathways. Using a multi-method approach, we assess factors affecting Ukraine’s forest SES and identify three key recovery pathways: (1) an economic focus through forest use for low-carbon recovery, including timber for reconstruction and small-scale energy production; (2) a “close-to-nature and close-to-people” approach, leveraging digital tools and innovations for climate-resilient recovery; and (3) anticipatory governance and socio-ecological innovation. Relying on a single pathway risks long-term vulnerability. We highlight that integrating recovery pathways creates synergies. Coordinated actions within Ukraine and from international actors are essential to enable a recovery aligned with “build back better” principles. Our findings offer valuable insights for Ukraine and other conflict-affected regions.

**Graphical abstract:**

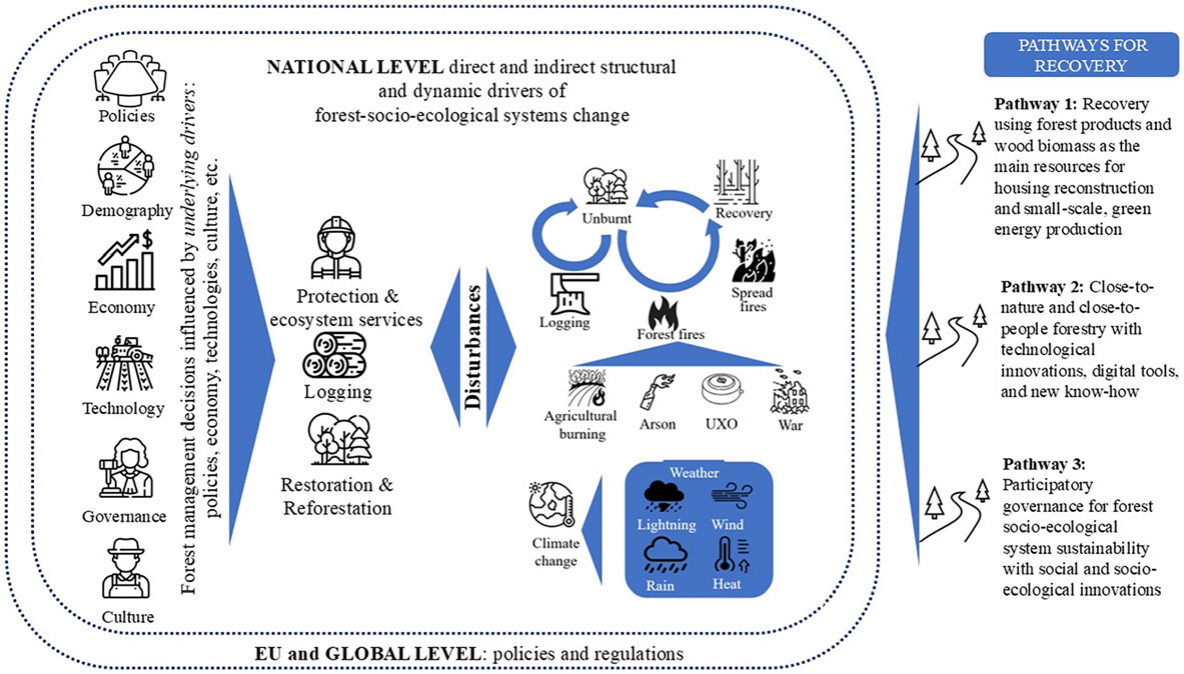

**Supplementary Information:**

The online version contains supplementary material available at 10.1007/s13280-025-02263-0.

## Introduction

Military conflicts have historically impacted natural ecosystems, including forests, affecting not only provision of their ecosystem services, but also well-being of forest-dependent communities and other users of natural assets. These conflicts disrupt decision-making structures, processes, and institutions, influencing the entire forest socio-ecological system (SES), which integrates ecological processes with sociopolitical and economic dynamics (as conceptualised, for example, by Folke et al. 2005; Ostrom 2009; Palomo et al. 2014; Sarkki et al. 2017; Melnykovych et al. 2018; Sarkki et al. 2019a). Post-war recovery of forest SES typically encompasses several key patterns including demining, forest restoration and reforestation, and involves different stakeholders and local communities, land-use changes, conservation, or even land abandonment (Walters 2017; Grima and Singh 2019; Rudel et al. 2020; Leal Filho et al. 2024a, 2024b; Meaza et al. 2024). These recovery processes often reshape the landscape and affect not only ecological functions but also social and economic dimensions, as communities rely on forests for resources and livelihoods. Recovery efforts can also provoke conflicts among land users, which can lead to significant changes in land tenure and its administration, affecting short-term and long-term sustainability (Unruh and Williams 2013; Vanegas-Cubillos et al. 2022; Flower et al. 2023). Recent research highlights that armed conflict not only disrupts ecosystems but also alters how people interact with nature and perceive environmental policies, as seen in cases of border militarisation (Nowak et al. 2025) and shifts in green preferences across Europe following the war in Ukraine (D’Ecclesiis et al. 2025). Against this backdrop, we analyse Ukraine’s forest socio-ecological systems, focusing on pre-war challenges, war-induced impacts, and possible recovery pathways across ecological, socio-economic, and governance dimensions. We place particular emphasis on resilience and alignment with global, EU, and national policy trends, recognising that while Ukraine draws inspiration from EU frameworks, its forest policies also reflect distinct national priorities and local governance contexts.

Indeed, lessons learnt from past conflicts suggest that recovery of forest SES is not an easy task. In Europe, after World War II, different countries adopted a combination of strategies to deal with post-war recovery challenges. Germany, Poland, and France, as well as Ukraine, implemented large-scale reforestation projects aimed at restoring timber supplies and preventing soil erosion (Gensiruk 2002). These efforts were primarily state-driven and aimed at stabilising the forest sector (Redo et al 2012; Forino et al 2015). In the Ukrainian Carpathian region, along with the excessive use of forests (in 1956–1960, the annual volume of timber harvested exceeded the average increment by almost double), extensive reforestation took place (Gensiruk 2002). Reforestation efforts with widespread planting of spruce (*Picea abies*), often on sites where the species does not naturally occur, led to increasing vulnerability to pests, pathogens, and drought (Kuemmerle et al. 2007; Keeton et al. 2013; Kholiavchuk et al. 2023).

After Ukraine’s post-independence period (i.e. after 1991), abandonment of marginal agricultural lands became increasingly common (Baumann et al. 2011). Many of these lands underwent natural regeneration, eventually giving rise to new forest types on previously unmanaged land (Kuemmerle et al. 2007). Natural forest recovery has been observed in several contexts of land abandonment, for example, in the Carpathian region (Smaliychuk et al. 2016). Similar, but war-related, patterns of natural reforestation have occurred in post-conflict regions such as Bosnia and El Salvador, where the cessation of agriculture and land-use discontinuation—often due to contamination—enabled forest regrowth (Le Billon 2000; Musa et al. 2017). In many of these cases, however, the socio-economic functions of forests remain limited, as the presence of landmines and unexploded ordnance (UXO) hinders forest management, timber harvesting, and outdoor recreation.

Post-war shifts in power relations have often led to institutional changes in forestry. The collapse of communist regimes in Eastern Europe in the late 1980s to early 1990s resulted in privatisation of forestland and creation of new governance systems in several countries, including Romania and Poland, with varying continuity of sustainable forest management (Knorn et al. 2013; Smaliychuk et al. 2016). Elsewhere in the world, lessons from Cambodia, Thailand, Bolivia, and Brazil also highlight the need for institutional changes. They call for strengthening forest governance through active community participation—beyond just formal land rights—as crucial elements in putting post-war forestry on a sustainable path (Le Billon 2000; Cronkleton et al. 2011; Jenke 2024).

A critical challenge in post-war recovery is contamination of forests by UXO, which severely restricts access to forests (OECD 2022; Leal Filho et al. 2024b). In Bosnia and Herzegovina, ten years after the war, large areas still remain contaminated, hindering recovery efforts and delaying the delivery of economic and social benefits from forest SES (Henig 2012; Musa et al. 2017; Baselt et al. 2023). In Croatia, Serbia, Armenia, and Ukraine, landmines also limit forest management and use. Also, forestland, wood and biomass often contain projectile fragments, making them unsuitable for use (WWF 2023; Nehrey and Finger 2024).

In Ukraine, ongoing war drives forest contamination and widespread forest fires due to continuous shelling and has significantly affected forest health and vitality and the provision of ecosystem goods and services (Pereira et al. 2022; Prins 2022; Irland et al. 2023; Shumilo et al. 2023; Zibtsev et al. 2023a; Leal Filho et al. 2024b; Matsala et al. 2024; Myroniuk et al. 2024). While the war in Ukraine began in 2014 and the natural ecosystems in the eastern part of the country have been affected since then (Milakovsky et al. 2025), this paper focuses primarily on the impacts of the full-scale Russian invasion which began in 2022. It reflects the sharp escalation in the scale of military actions and their environmental consequences and elaborates post-war recovery pathways. The war in Ukraine has triggered far-reaching geopolitical, economic, and humanitarian disruptions, with cascading effects on institutional capacity and land use, forest ecosystems and agriculture, impacting global food security (WWF 2023; Nehrey and Finger 2024). Ukraine is now among the most heavily mined countries globally, with an estimated 139,000 square kilometres—around 20% of its territory—contaminated by landmines and unexploded ordnance (United Nations 2025). This widespread contamination threatens civilian safety, impedes land use and hinders efforts to restore ecosystems. For forests, the presence of numerous mines has led to restricted access and limited logging operations, limited protection of forest from fires and insects and diseases, restraining reforestation and ecosystem restoration efforts, and the provision of non-wood forest products (e.g. mushrooms and berries), as well as recreational activities in woodlands (Musa et al. 2017; Flamm and Kroll 2024; Hryhorczuk et al. 2024; Leal Filho et al. 2024a). The war has resulted *“in the worst crisis in the history of Ukraine’s forest sector”* (Zibtsev et al. 2023; Mitrofanenko et al. 2024). According to UNEP (2022), there is an urgent need to avert the cascading impacts of war and build resilience in Ukraine, which “*is now facing a compounded, multi-dimensional environmental crisis that has either exacerbated existing issues or added new ones*” (UNEP 2022, p. 6). Indeed, “*the environment continues to be the silent victim of armed conflicts worldwide*” (UNEP 2019) and with over 120 armed conflicts ongoing globally (ICRC 2024), significant efforts are required to ensure socio-ecological recovery.

This paper explores Ukraine’s forest SES prior to the war (Sect. “Baseline Assessment: Contextual review of Ukraine’s forest socio-ecological systems”), by applying the multi-method approach presented in Sect. “Materials and Methods”. It explains the root causes of disturbances in forest SES and impacts of the war, and the resulting crisis in Ukraine (Sects. “Nature of the Crisis” and “Scope of the crisis”). It then identifies pathways for the recovery of forest SES (Sect. “Resolution: Pathways for recovery”) and discusses how these sustainability pathways align with current policies and trends in the EU and globally (Sect. "Discussion"), emphasising the need for synergies across diverse initiatives to ensure resilience despite ongoing geopolitical pressures.

## Materials and methods

Our study employs a multi-method approach to analyse socio-economic, environmental, and governance drivers and pressures on Ukraine's forest SES. This analysis adapts the analytical framework proposed by Bergström et al. (2016): (1) system dimension, (2) system scope, and (3) system resolution to the Ukrainian context. This enables examination of the crisis caused by the war as (1) the nature of the crisis (corresponding to the system dimension), (2) scope of the crisis (corresponding to the system scope), and (3) resolution of the crisis (corresponding to the system resolution) (see Table 1).

**Table 1:** Key dimensions of Nature of the Crisis, Scope of the Crisis, and Resolution of the Crisis and their analytical explanations. “Root causes” refers to systemic drivers affecting Ukraine’s forest socio-ecological systems, as part of the adapted analytical framework from Bergström et al. (2016). The analytical framework based on three system dimensions. Each aspect can consist of one or more steps and each step details the analysis and sources of information

Dimensions	Steps	Type of analysis	Sources of information
(1) Nature of the crisis	Definition (pre-war challenges and war-induced crises)	Literature review (scientific publications and grey literature)	Web of Science; Google Scholar; government websites
		Expert interviews	Ukrainian researchers, government officials and representatives of NGOs, n = 7
		Forest policy document analysis Data from National Statistics	IUFRO report from Forum on Ukraine Forest Science and Education: Needs and Priorities for Collaboration (Zibtsev et al. 2024a); FOREST EUROPE report on Ukraine’s forest recovery (Forest Europe 2023); World Bank reports on Ukraine (World Bank 2020; 2024); OECD report on Ukraine (OECD 2022); State Forest Management Strategy of Ukraine until 2035 (SSSU 2024); and FAO Forestry Support Strategy for Ukraine 2023–2027 (FAO 2023) Official information on forests of Ukraine of the State Forest Resources Agency of Ukraine (2025)
(2) Scope of the crisis	Identification of root causes of disturbances in Ukraine’s forest socio-ecological systems	Literature review (scientific publications and grey literature)	Web of Science, Google Scholar, survey of peer-reviewed publications, Ukrainian government and NGOs reports
	Assessment of impacts	Forest cover change	Official data from Ecozagroza online platform; data from SFRA; website of the Ukrainian state forest management planning association "Ukrderzhlisproekt"; and—Data of the Remote Sensing-Based National Forest Inventory (Myroniuk et al. 2023)
		Fires and area burned	State Emergency Service of Ukraine (SESU) Regional Eastern Europe Fire Monitoring Center (REEFMC 2025) Landscape Fires Advisory Bulletin
		Society	ZOI Environmental Network, Copernicus Dynamic Land Cover map, Emerald Network, grey literature, and expert interviews (n = 7)
		Economy	Forest statistics, UKRstat webpage
		Governance	Scientific publications, the IUFRO report on the Ukraine Forum *“Forest Science and Education: Needs and Priorities for Collaboration”* (Zibtsev et al. 2024a), and results of the discussion held during the special session at the IUFRO World Congress on Ukraine’s forest (2024); Ukraine’s forest policy regulations and amendments to law due to martial order (2022–2024); reports of international organisations and programmes (e.g. FAO, WB, WWF, UNECE)
(3) Resolution of the crisis: Recovery pathways	Evaluation of actions/ responses in place; suggestion of recovery pathways	Literature review and current policies; expert interviews Thematic analysis (QDA Miner Lite)	Web of Science; Google Scholar Reports: World Bank 2020; OECD 2022; FAO 2023; Forest Europe 2023; SSSU 2024; World Bank 2024; Zibtsev et al. 2024a Interviews with Ukrainian researchers, government officials and NGO representatives (n = 7)

The additional integration of a literature review with insights from expert interviews enables us to examine both pre-war challenges and war-induced impacts on Ukraine’s forests between 2010 and 2025. This approach and time frame allowed us to develop plausible recovery pathways grounded in both empirical evidence and expert perspectives.

The literature review provides a foundational understanding of the forestry challenges and potential recovery strategies in Ukraine. Using Web of Science and Google Scholar, we conducted searches with the following keywords: "forest”, "forestry”, "forest sector”, "war”, "landmine*”, "UXO”, "recovery”, "socio-ecological”, "forest governance”, "forest policy”, "biodiversity”, "timber”, "harvesting”, "sustainable forest management”, "Ukraine”, and Boolean combinations of these.

We reviewed titles and abstracts to identify studies focused on Ukraine’s forest economy, forest-dependent communities, social dynamics, biodiversity, timber production, harvesting practices, sustainable forest management, governance, ecological resilience, stakeholder engagement, and post-war recovery. Additional context was drawn from grey literature such as the IUFRO Report on Ukraine Forest Science and Research, the State Forest Resources Agency of Ukraine (SFRA), and the FAO Forestry Support Strategy for Ukraine (2023–2027).

This line of reasoning shaped the design of interview questions and provided a comparative basis for interpreting stakeholder interviews (Appendix SI 1-3).

We conducted semi-structured interviews with seven Ukrainian forestry experts (n=7), including researchers, government officials, and representatives from forest-related NGOs. These participants were selected for their direct involvement in forest recovery efforts. The interviews, conducted in 2024, addressed governance, ecological impacts, and socio-economic pressures, allowing us to capture diverse viewpoints essential for accurately reflecting on the complexities in forest SES.

1. **Nature of the crisis**
  This dimension of our analysis defines key challenges and governance issues through the literature review and expert interviews. Selected experts, who are central to Ukraine’s forest recovery efforts, offered in-depth insights into current challenges and strategic opportunities. Their perspectives were instrumental in identifying nuanced governance and operational issues that affect the forest sector’s resilience and sustainability (Table 1).
2. **Scope of the crisis**
  The scope dimension establishes the boundaries of the crisis, addressing *what, where, when,* and *how much* has been affected. This step enabled us to uncover the root causes impacting Ukraine’s forest SES, particularly those associated with war and institutional factors. Our analysis encompassed spatial and temporal impacts, drawing on data such as satellite imagery from the ZOI Environmental Network, land-cover data from the Copernicus Dynamic Land Cover Map, and data from the State Emergency Service of Ukraine (SESU) and the Regional Eastern European Fire Monitoring Center (REEFMC). We also examined socio-economic factors, including the increased demand for timber due to war-related reconstruction needs, to understand the most acute pressures on Ukraine’s forest sector (Table 1).
3. **Resolution of the crisis: Developing recovery pathways**
  The resolution dimension addresses the how, where, and when of implementing post-war recovery pathways for Ukraine’s forest sector. We developed strategic interventions aimed at building resilient SES, strengthening governance frameworks, and promoting participatory decision-making through social innovations. To guide the outline of these pathways, we followed the approach proposed by Beland Lindahl et al. (2015), which emphasises that sustainability pathways should emerge from concrete, context-specific challenges. Drawing on expert interviews and literature, we therefore identify recurring themes focused on three key areas of challenge for obtaining sustainability goals, according to Beland Lindahl et al. (2015, 2017): socio-economics and policy, technological innovations, and environmental and governance innovations. These may include socio-economic pressures (e.g. rising timber demand, illegal logging), environmental disturbances (e.g. climate change-related impacts, armed conflict or war-related land degradation, wildfires, biodiversity loss), and institutional weaknesses (e.g. governance weaknesses, limited transparency, power asymmetries). Using QDA Miner Lite, we applied thematic analysis (Adu 2019) to group literature review results and experts’ responses along these themes (Appendix SI 2; SI 3). These were then used to formulate the three recovery pathways presented in Section “Resolution: Pathways for recovery”.
  Drawing from both published research and document analysis in combination with expert interviews and case studies, we provide a comprehensive outline of sustainability pathways to address Ukraine’s forest socio-ecological challenges. This cross-verification across different types of evidence and perspectives helps to ensure that the identified pathways are not only relevant and contextually grounded but also offer insights that could apply to comparable forestry settings elsewhere. Despite the limited sample size of interviews, our multi-method approach ensures the credibility and validity of research findings.

## Results

Ukraine’s forest SES are under severe strain, with the war intensifying ecological, socio-economic, and institutional challenges.

### Baseline assessment: Contextual review of Ukraine’s forest socio-ecological systems

Forests in Ukraine cover 10.4 million hectares (15.9% of the country) placing it ninth in Europe by forest area (SFRA 2023a). Ukraine’s forests are predominantly publicly owned (86.5%) and under the management of the State Specialized Forest Enterprise “Forests of Ukraine”. The enterprise is not financed by the state budget and independently sustains its economic activities. It consists of a central office, 9 regional offices and 144 branches. Around 13% of forestland is communal and managed by communal forest enterprises and only 0.1% of forests are small-scale private property (SFRA 2024a). The State Forest Resource Agency (SFRA) is responsible for elaboration and implementation of state policy for forestry and hunting, forest management planning, issuing harvesting permissions and international activities, inter alia, but not directly for forest management. In Ukraine, the role of private industry in the forest sector is limited primarily to timber harvesting operations—typically carried out by small contractual businesses—and wood processing activities, rather than direct involvement in forest ownership or long-term forest management.

Forest distribution varies regionally, with the highest forest cover in the Carpathian Mountains (52%) and Polissia regions (40–42%) in west and north-west Ukraine, respectively. In Ukraine, forests are vital for biodiversity, climate regulation, and supporting local livelihoods, as well as for providing timber and other ecosystem services (Melnykovych et al. 2018; Soloviy and Melnykovych 2019; Nijnik et al. 2020, 2021). Productive forests dominate the north-west and west of the country, while in the steppes of central and southern Ukraine, forest cover is sparse (5–12%), yet shelterbelts are critical in protecting larger agricultural fields from windblown erosion, providing habitats for biodiversity at smaller scales, and accommodating traditional land uses (Yukhnovskyi et al. 2021; Yaroshchuk et al. 2023).

Ukraine’s forests are categorised for various uses: 38% are designated as commercial, 33% as protective, 15% for recreational purposes, and 14% as natural reserves for scientific and cultural preservation (SFRA 2024c). The estimated timber volume stands at 1736 million m^3^, with an annual increment of 35 million m^3^ and an annual harvesting rate of 20 million m^3^. Forest management is conducted by professionally trained foresters. According to the Forest Stewardship Council (FSC), in October 2021, Ukraine certified 3.72 million hectares of forests, with 118 forest management and chain of custody (FM/CoC) certificates issued (Vacik et al. 2020).

Forestry contributes approximately 0.3% to Ukraine’s GDP. The sector saw substantial growth in 2022, with timber sales reaching UAH 21,583.4 million (USD 584.4 million), primarily driven by exports of unprocessed timber (SFRA 2024a). In the period 2010–2023, of the total volume of harvested timber, 41% was used for industrial purposes and 48% for firewood, primarily for heating and energy (SSSU 2024). Beyond economic contributions, forests provide essential ecosystem services and support around 36,000 jobs (SSSU 2024). Forests also play a critical role in the country’s rural livelihood (FSC 2024; Melnykovych et al. 2016). Local communities rely on forest resources, e.g. for employment in the forest industry or tourism, and especially for non-timber forest products (NTFPs) such as berries and mushrooms (Vacik et al. 2020), which have seasonal and cyclical yields (Melnykovych et al. 2017; Sarkki et al. 2019a, 2019b).

The forest sector in Ukraine does not fully meet high and changing societal expectations (Melnykovych et al. 2018). Issues such as illegal logging, cases of corruption, low consideration of multiple ecosystem services other than timber in decision-making, and a lack of transparency in forest management have raised concerns among scientists, NGOs, media, and local communities (Soloviy et al. 2017). While institutional transformations have begun and positive trends are observed, the rules of the game have not changed significantly (Nijnik et al. 2020, 2021). Despite the introduction of open consultations and public boards to advise regional and central offices of the SFRA, forest governance in Ukraine is predominantly centralised and features a rigid top-down approach (Sarkki et al. 2019a, 2019b; Nijnik et al. 2020). Decentralisation of forest management—giving local forest enterprises more control over their operations and finances to ensure profitability—has not yet been fully realised. Efforts to transfer administrative and financial independence to these enterprises remain incomplete (FAO 2023; Poliakova and Abruscato 2023). Lack of policy coordination and weak cross-sectoral collaboration are evident (Soloviy and Melnykovych 2019; FLEG 2010). Although market instruments and forest certification efforts have increased, there is a need for greater involvement from government, civil society, and private-sector actors (Nijnik et al. 2021; FAO 2023; FSC 2024).

Forest policy has increasingly become aligned with European Union standards, emphasising sustainability, biodiversity conservation, and compliance with EU environmental directives (Lesiuk and Soloviy 2024). The Forest Code of Ukraine, established in 1994 and last amended in 2024, serves as the primary regulatory framework for forest management. However, its implementation has been constrained by limited resources (Dykan and Khoroshko 2022) and institutional capacity, thereby hindering the practical uptake of sustainable forest management (Dubovich et al. 2019). Current policy priorities—e.g. the State Forest Strategy (2023)—include decentralising forest management, promoting public–private partnerships, dividing governance and business power among different actors, and advancing a green economy approach in line with the EU Association Agreement (Khvesyk et al. 2019).

While the Baseline Assessment outlines the foundational characteristics of Ukraine's forest socio-ecological systems, the next section (3.2.) explores the multidimensional challenges that have compounded these vulnerabilities, both before and during the war.

### Nature of the crisis

Following on from Section “Baseline Assessment: Contextual review of Ukraine’s forest socio-ecological systems”, which outlines the general context of Ukraine's forest socio-ecological systems, this subsection focuses on specific pre-war challenges which have amplified vulnerabilities and limited the sector's capacity to respond to future crises.

#### Pre-war challenges

Building on the Baseline Assessment (3.1.), this subsection focuses on challenges faced by Ukraine’s forest sector before the full-scale invasion in 2022. Stemming from the beginnings of the war in 2014, many systemic pressures and sectoral dynamics persisted until 2022 under relatively stable governance and market conditions.

Ukraine's forest sector faced significant challenges even before the current conflict, including the impacts of climate change, land-use changes, and institutional and financial constraints. Limited participatory governance and cases of corruption, primarily associated with timber logging, further exacerbated these issues (Soloviy et al. 2017; Denisova-Schmidt et al. 2019; Nijnik et al. 2020; Gisladottir et al. 2021). These factors contributed to a sometimes-negative societal perception of forestry and foresters (Soloviy et al. 2017).

Between 2019 and 2022, climate change and associated natural disturbances caused the dieback of approximately 800,000 hectares of forest, including 420,000 hectares of pine forests severely affected by bark beetle infestations (Zibtsev et al. 2023a, 2023b). Since the early 2010s, Ukraine has experienced extreme weather events with more frequent heatwaves and droughts, and snowless winters creating conditions highly favourable for large-scale fires (Zibtsev et al. 2023a, 2023b). The fire seasons of 2014, 2015, and 2020 were particularly severe, with human activities contributing to widespread wildfires across the country. The fire season of 2020 was the worst in Ukraine’s history, with large fires affecting the Chornobyl Exclusion Zone (67,000 ha), Zhytomyr oblast (43,000 ha, territory comparable to a province or canton; a first-level administrative unit), Kharkiv oblast (8000 ha), and Luhansk oblast (39,500 ha). In July 2020, fire in Eastern Ukraine destroyed more than 730 houses, resulting in 17 fatalities and 861 injuries (Soshenskyi et al. 2023). In 2020, forest fires in the north-west Polissja region affected 7 villages, damaging 82 houses. In north-east Kharkiv oblast, forest fires destroyed 22 private houses (Soshenskyi et al. 2023). In the Chornobyl Exclusion Zone, wildfire damage to natural ecosystems was estimated at approximately USD 205 million (Soshenskyi et al. 2023).

Institutional challenges, such as declining state funding and an overreliance on state control, have hampered management operations and innovations in the forest economy (Nijnik and Oskam 2004; World Bank 2020). Problems stemming from the separation of forest management from policymaking and lack of transparency have led to increased risks of corruption, illegal logging, limited cross-sectoral collaboration, and outdated information systems, all of which have hindered the sector’s sustainability (Soloviy et al. 2017; Nijnik et al. 2020, 2021), further eroding public trust (Melnykovych et al. 2018; Sarkki et al. 2019a, 2019b; Nijnik et al. 2021; Hrynyk et al. 2023).

Recent reforms in the forest sector (CMU 2021) are aimed at addressing existing challenges by prioritising sustainable management practices, including close-to-nature forest management approaches. These reforms also focused on increasing transparency through more active public participation and enhanced collaboration of different stakeholders through consultations and public dialogues. Digital tools were also introduced such as auctions in the timber market and the implementation of a tracking system for harvested timber to reduce illegal logging and trade (Soloviy et al. 2017; Nijnik et al. 2020). Reforms also led to the separation of policymaking and management functions through the creation of the specialised forest enterprise "Forest of Ukraine", responsible for operations and forest management, and the aforementioned State Forest Resource Agency, focused on policy (State Forest Strategy 2023). The reforms implemented by the Ukrainian Government (CMU 2021) aimed to enhance community involvement in forest management, governance and use, but they remain limited in scope and impact. Expert 3 noted “*these reforms primarily emphasised increasing economic efficiency through too centralised management functions, which raises the risk of disconnection from local communities*”. However, innovations in forest governance, with new social initiatives emerging, have been observed in recent years, marking a positive shift towards more active engagement of civil society actors in reconfiguration of social practices in addressing contemporary challenges (Melnykovych et al. 2018; Nijnik et al. 2020).

Significant positive advancements have been observed in the promotion of information technology and innovative digital platforms in the sector, enabling online auctions and societal monitoring of timber harvesting and trading. Importantly, there has been growing recognition of the need to enhance integrative, multifunctional forestry and certify non-timber ecosystem services (FSC 2024).

#### War-induced crises

The Russian full-scale war in Ukraine is exacerbating challenges (FAO 2023). As announced at COP29 in November 2024, after 1000 days of war, costs in environmental damage were estimated at USD 71 billion, and led to the equivalent of some 180 million tons of carbon emissions (Ministry of Environmental Protection 2024). Approximately one-third of Ukraine’s forests, spanning 2.9 million hectares, have been impacted (Poliakova and Abruscato 2023), with around 1.3 million hectares contaminated by UXO, rendering these areas hazardous for humans, animals, and the ecosystems (SFRA 2024a). Protected areas suffer extensive damage, with large-scale fires reported in regions experiencing active military operations (SFRA 2024a). As of June 2023, war-related economic losses in the sector were estimated at USD 530.8 million (SFRA 2024a).

The war has also significantly disrupted forest restoration efforts announced in 2021, such as implementation of the Green Country project, which aims to plant 1 million hectares over 10 years to increase forest area (Decree 2021, Poliakova and Abruscato 2023). Tree planting decreased from 54,500 hectares in 2021 to 36,900 hectares by early 2023 (SSSU 2024; Green Country Project 2021), while timber demand increased.

The war severely impacts forest research, management and education with many students and researchers forced to relocate. This leads to significant disruptions in forest research capacity and impacts forest professionals. The Ukrainian forest sector now faces a severe shortage of skilled professionals, with 2390 foresters mobilised for military service. As of mid-2024, the State Forest Agency of Ukraine reports that the war has resulted in the deaths of 128 foresters, the loss of 524 pieces of forestry equipment, and the elimination of 2738 workplaces (Zibtsev et al. 2024a).

Direct damage to SES from warfare is severe (UNEP 2022), and biodiversity and soil health are impacted by extensive fires and soil erosion. The destruction of shelterbelts (windbreaks) around agricultural fields (Elbakidze et al. 2024) is significant, since they were historically established in the central–east of Ukraine on agricultural lands to play a crucial role in agroforestry systems, primarily reducing wind speed to mitigate erosion, protect crops, and retain soil moisture (Yukhnovskyi et al. 2021). Protective and regulatory forest functions are especially important in semi-arid regions where they help prevent soil degradation and conserve water. In changing climate conditions featuring increased drought, these shelterbelts have become vital nature-based solutions for preserving soil fertility and ensuring food security. However, during the war, many shelterbelts have been repurposed for warfare and become strategic areas for military operations or serve as burial sites (Irland et al. 2023). This leaves the affected regions even more vulnerable to environmental risks.

In areas such as the Ukrainian Carpathians, where forests are not directly impacted by the war, climate-driven disturbances like wildfires, pest outbreaks, and other ecological stressors are worsening forest health (Zibtsev et al. 2023a, 2023b). These forests also face additional pressures due to the relocation of businesses and increased numbers of people residing in the region due to internal displacement from the east of the country (Melnykovych et al. 2024a; Mitrofanenko et al. 2024; Wypych et al. 2024).

The challenges described provide the foundation for understanding the full scope of the crisis, which is examined in the following Section (3.3.) through the lens of structural drivers and their far-reaching impacts.

### Scope of the crisis

#### Root causes

**Fig. 1:**
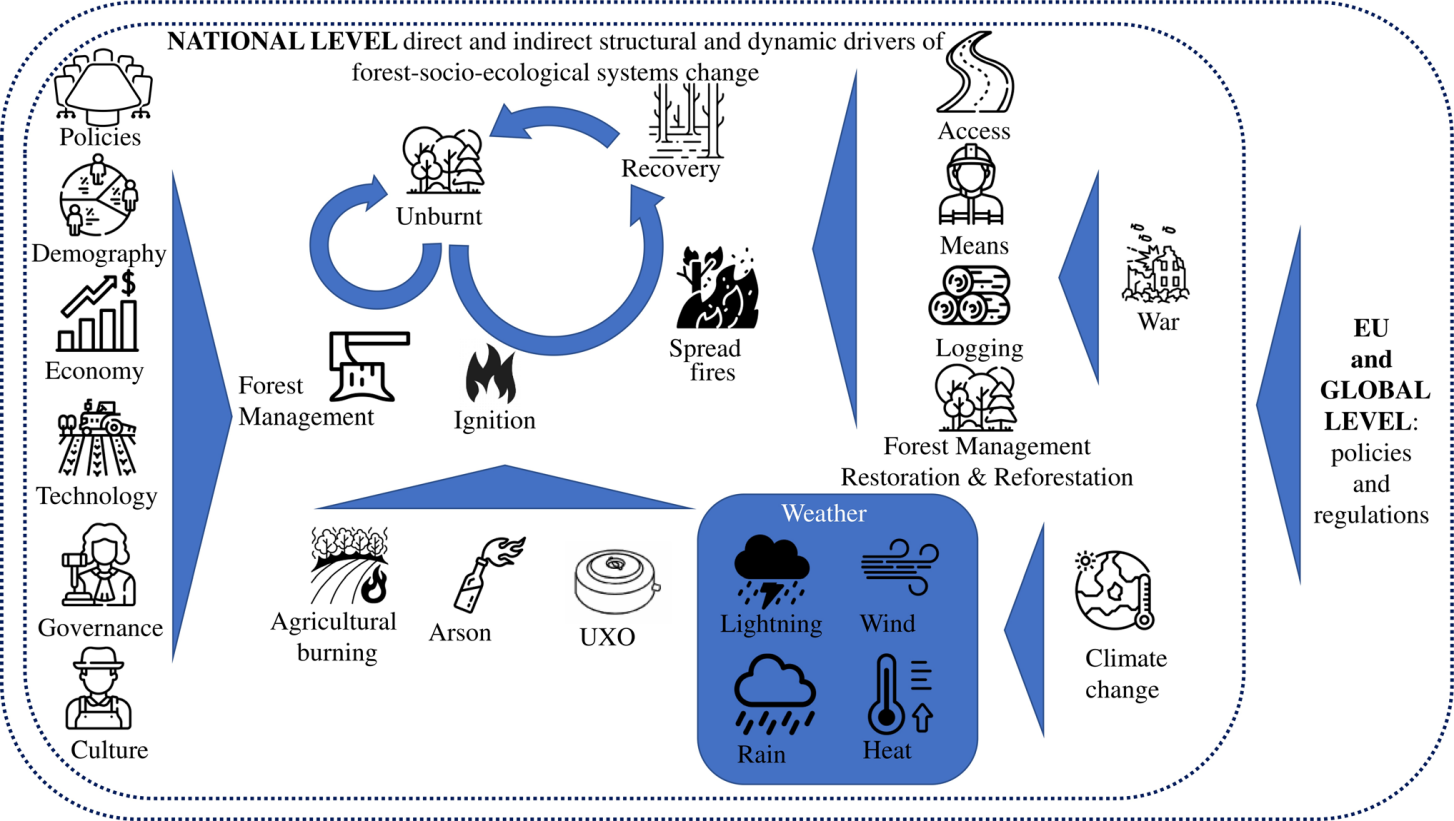
Root causes highlight the structural and systemic drivers of change within Ukraine's forest socio-ecological systems. *Note*: The figure emphasises fire as a dominant disturbance process, which, in pre-war times, was primarily relevant to fire-prone systems, such as Scots pine (*Pinus sylvestris*) forests in Ukraine’s north (e.g. the Polissia region and the Chornobyl Exclusion Zone). Other forest types, such as those in the Carpathian region, were more strongly influenced by wind, insects, and pathogen disturbances. During the war, however, fire has emerged as a key disturbance factor, as illustrated in the figure. Its impact on forest SES is most severe in frontline regions, where forest fires are nearly unstoppable. This figure illustrates the complex interactions shaping Ukraine’s forest socio-ecological systems in the context of war. On the left side, key underlying societal drivers—including policies, demography, economy, technology, governance, and culture—represent structural forces that indirectly influence forest management decisions. These drivers, shaped by national and global dynamics, determine how forests are valued, governed, and restored. In the centre, a range of disturbances—such as war, unexploded ordnance (UXO), arson, agricultural burning by local communities or family-scale farms, and climate-induced events—disrupt forest systems, leading to altered trajectories of degradation, persistence, or recovery. These disruptions, in turn, shape management responses such as logging, protection, and reforestation

The results of our analysis provide evidence that root causes of the crisis in Ukraine's forest SES are driven by a complex interplay of structural and systemic factors, with war being the main one (Fig. 1). Ukraine's forest SES are shaped by natural processes (e.g. climate change), land-use changes, management decisions (which could be unsustainable, see Section 4.2.2.) and anthropogenic disturbances (e.g. wildfires). Forestry practices, influenced by underlying natural conditions, demography, politics, economy, technology, governance, and societal expectations affect SES. Human actions, such as unmanaged agricultural burnings or illegal logging, and more recently and critically, warfare, war-related arson, and shelling—with often long-lasting presence of UXO—have significantly intensified pressures on forest SES. Military actions, featuring explosives, rockets and drone attacks, and large vehicle movements, spark fires with devastating impacts (Zibtsev et al. 2022; CEO and Zoï 2024) . Climate change exacerbates these challenges by altering weather patterns, affecting forest growth, and raising further wildfire risks. Increasing forest fires from continuous shelling, human-caused ignition, contamination of land with UXO, and other factors pose significant threats. They also hinder access to firefighting resources and complicate fire management, including fire prevention and suppression efforts (Soshenskyi et al. 2021a).

Forests in the northern, eastern, and southern regions have been particularly vulnerable (SFRA 2024a, 2024b). The warfare has led to widespread habitat destruction, further threatening biodiversity and exacerbating the already challenging task of sustainable forest management. Experts 1, 4, and 6 stated that fire ignition and spread are not primarily influenced by natural factors such as lightning and wind but shaped by continuous shelling, as well as human decisions related to land use and forest management, such as arson and burning of agricultural fields.

Importantly, “*the ongoing war has led to significant increase of demand for timber*” (Expert 4), primarily driven by the need for wood for military fortifications, and for energy due to shortages in Ukraine. Interviewed experts anticipate a further increase in timber demand, driven by the need to support reconstruction efforts, comply with EU policy legislation for achieving net zero targets (Plan for the Recovery of Ukraine 2024), and promote the shift to “green energy sources” highlighted by Ukraine's government (Lugano Declaration 2022).

#### Impact

##### Forest fires and biodiversity loss

**Fig. 2:**
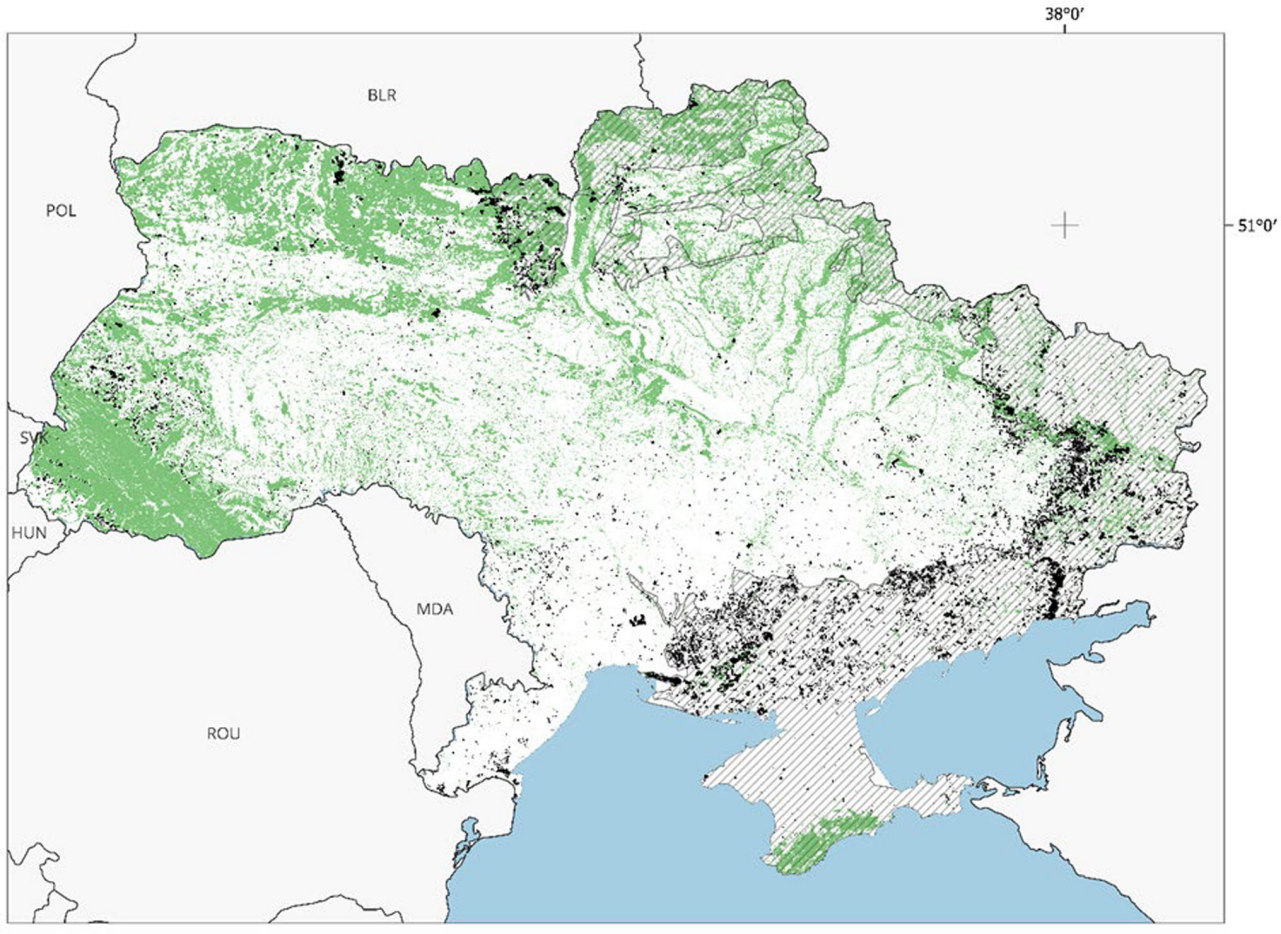
Forest and landscape fires across Ukraine from 1 January 2022 to 31 August 2024. *Note*: Wildfires affect different types of land cover, including forests, grasslands, and agricultural lands, resulting in environmental impacts such as loss of biodiversity, soil degradation, and contribution to air pollution, including GHG emissions. Cumulative fires are shown on the map, with fires marked in black, forests in green, water bodies in blue, and active war or occupied zones (as of June 2024) in grey

The proximity of populated areas to forests, combined with intensive agricultural activities, increases risk of wildfires. Extreme droughts in some regions, exacerbated by climate change and often linked to heatwaves, increase their frequency and scale (Soshenskyi et al. 2021a). Military activities are also responsible for a significant number of forest fires (Zibtsev et al. 2023a). The Russian war against Ukraine, which started in 2014 (with the full-scale invasion launched by the Russian Federation on 24 February 2022) has escalated both the number and extent of wildfires, especially in the eastern, southern, and central-northern regions of the country (Fig. 2).

The war has caused a significant threat to biodiversity. Approximately 900 protected areas—including Ramsar sites, river deltas, and critical Steppe ecosystems—have been affected by military activities such as shelling, bombing, and oil spills as well as soil pollution with heavy metals (Solokha et al. 2023). Since 2022, landscape fires in protected areas have burned 329 672 hectares, with 44.2% in territories occupied by Russia and within a 30-km frontline buffer zone (Zibtsev et al. 2022, 2023a, 2023b, 2024a, 2024b; REEFMC 2024).

**Fig. 3:**
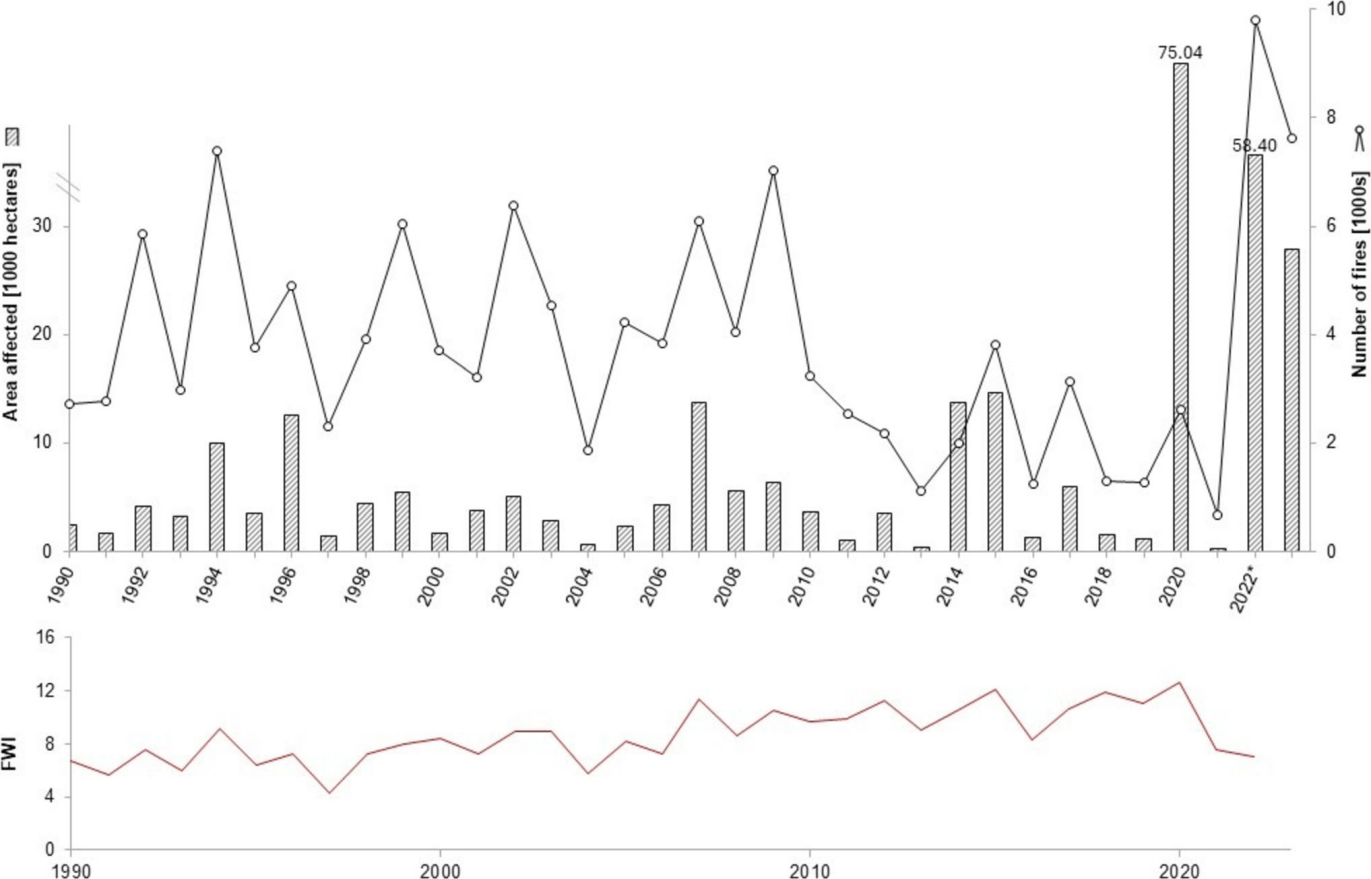
Forest fires in Ukraine (1990–2023). *Note*: The figure shows the number of forest fires (line, in thousands) and the area affected by forest fires in thousands of hectares (bars). Data for 2000–2021 are based on official government statistics (SSSU, 2000–2021), while 2022–2023 data were calculated by Zibtsev et al. (2023a, 2023b, 2024a, 2024b) using remote sensing methods of REEFMC (2025). Note the change in scale for the area affected by fires. The inset box displays the Canadian Fire Weather Index (FWI), which, according to the European Forest Fire Information System (EFFIS 2025), has shown a doubling trend since 1990, based on SSSU 2024 data

The full-scale war has significantly exacerbated environmental damage, with fires resulting from active combat operations thus far affecting 1.3 million hectares. Between the start of the full-scale war and August 2024, 115,700 hectares of forest within active military zones and occupied territories were affected by fires, accounting for approximately 90% of all forest fires in Ukraine during this period (Zibtsev et al. 2023a, 2023b, 2024a, 2024b; REEFMC 2024). Fire connectivity, fuel availability, and access to firefighting (which in Ukraine is often limited) are important preconditions for governing landscape fires. However, from 2010 to 2023, the country lost an average of 5000 hectares of forest annually due to wildfires (plus 14,000 hectares due to pests, diseases, and extreme weather conditions) (Fig. 3).

Landscape fires over more than two years of full-scale war in Ukraine have released nearly 6 million tonnes of carbon into the atmosphere from burning organic biomass, including forest, and resulted in over 15 million tonnes of greenhouse gas (GHG) emissions (De Klerk et al. 2024). Long-term impact projections indicate that biomass losses from damaged forests in Ukraine due to the war might result in 11-18 million tonnes of additional carbon emissions, nearly 85% of these projected to originate from forests located in frontline areas north-east of Ukraine (Bun et al. 2024; De Klerk et al. 2024).

##### Socio-economic and market dynamics in forestry

The war has inflicted substantial socio-economic impacts and market challenges in Ukraine. In 2022, its GDP saw a historic 29% drop, primarily driven by widespread damage to industrial infrastructure and natural capital, including forests (Rawtani et al. 2022). As of November 2024, due to warfare, 20 forest enterprises mainly in east-north of Ukraine have suspended economic activities, including two in Donetsk oblast, three in Zaporizhzhia oblast, seven in Luhansk oblast, two in Kharkiv oblast, and six in Kherson oblast (SFRA 2024b). Forest area of 0.5 million hectares in territories liberated from Russian occupation requires demining (SFRA 2024a). Nearly 0.8 million hectares of forest land is under occupation and information about these forests is missing. 20% of Ukraine’s nature reserves, encompassing 812 protected areas, are significantly damaged (Ministry of Environmental Protection 2023) and not suitable for use for recreational, research and educational purposes.

**Fig. 4:**
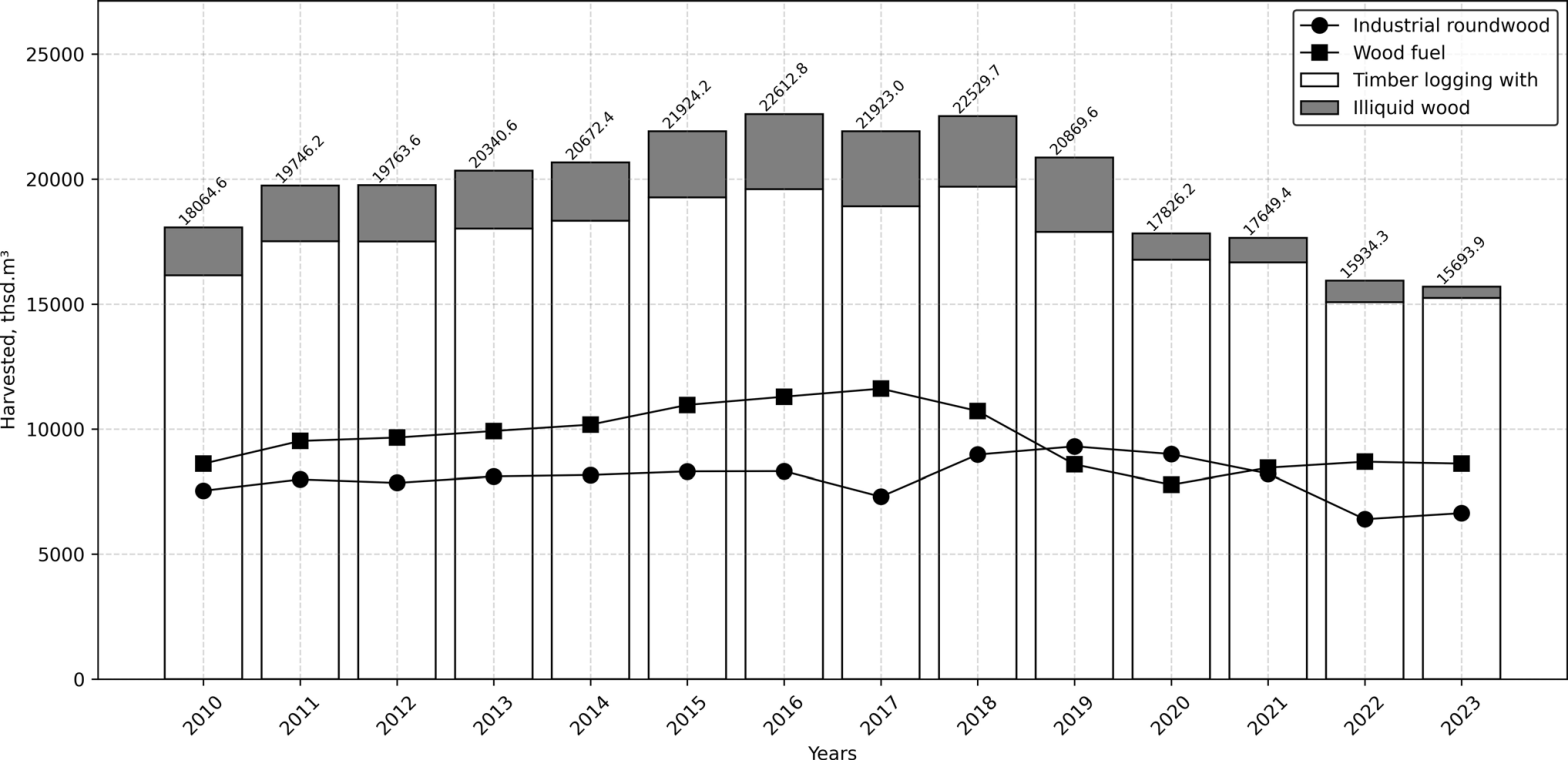
Dynamics of timber harvesting in Ukraine (2010–2023). *Note* Illiquid wood refers to wood that is difficult to sell or trade due to its poor condition or other factors

Logging volumes remained relatively stable from 2010 to 2021, with an average of 20.7 million m^3^ and maximum variations up to 15% range. However, in 2022 and 2023, when Russia's full-scale war against Ukraine began, harvesting volumes decreased by 23–24% (Fig. 4, (SSSU 2024)). Because most forests are concentrated in the northern and western regions of Ukraine, forest management is still possible, despite the war. In the south and east of the country, the main part of which is under occupation, forests (covering an area of approximately 0.8 million hectares or 7.7% of the territory) have no significant commercial value. In the territories that were previously temporarily occupied (north Ukraine), economic activities have been partially restored, except for the area of 0.5 million hectares that requires demining (SFRA 2024a).

Economic pressures on the forest sector are exacerbated by challenges in wood processing and timber marketing due to limited electricity supply since the war started, disrupted logistics, increased fuel prices, and a decreased area of FSC-certified forests.

Timber production has faced significant strains with a marked shift in 2022 towards fuel wood, which accounted for 61.7% of total timber sales (SFRA 2023). This trend reflects a rising demand for heating and burning wood to replace fossil fuels. Expert 6 noted that “... '*fuel wood' includes not only firewood for household use but also fuel wood designated for both industrial and non-industrial applications within industry”.* While fuel wood sales stabilised at 57.8% in 2023 (SFRA 2024a), the continued dominance of this segment points to market disruptions and underscores the need for strategic realignment. Market access challenges also persist, especially for sustainably managed forest products that lack consumer premiums, and the sector suffers from an absence of entrepreneurial development for non-timber forest products, despite the high value of such resources in some regions (FAO 2023).

Restricted forest access, increased fuel wood demand, and illegal logging have escalated. Illegal timber logging volumes in 2023 exceeded pre-war levels (SFRA 2024a), particularly in southern and eastern regions, where logging also emerged to support defence efforts and satisfy the immediate energy demands of local populations as a result of the destruction of energy infrastructure. Results from a survey conducted in late 2022 by ForestCom (2022), involving 51 respondents from five stakeholder groups—civil society activists, NGO environmental experts, forestry workers, wood processing workers, and institutional environmentalists—indicated that 66.7% believed the war had a significant impact on the daily operations of state forestry enterprises, flagging disruptions across the sector. Expert 6 highlighted that Ukraine’s forestry faces “*convergence of socio-economic and market challenges requiring sustained intervention, market monitoring, and a green recovery strategy that addresses immediate needs while paving the way for sustainable forest management and bioeconomy transitions*” (Expert 7).

##### Forest governance and management

Russia’s war in Ukraine necessitated the substantial refinement of Ukraine’s forest policy and strategic goals. The updated *State Strategy of Forest Governance of Ukraine until 2035* includes new provisions aimed at restoring forest management in areas contaminated by explosive ordnance and at rehabilitating forests damaged by armed conflict. However, forested areas are not designated as a priority for demining efforts, limiting the practical effectiveness of these provisions and rendering much of this policy declarative rather than actionable. Only 0.1% of the required demining area, or 232 hectares, has been addressed by the State Specialized Forest Enterprise “Forests of Ukraine”, indicating significant gaps in operational capacity and financial support for demining efforts. Voluntary forest certification, i.e. from the Forest Stewardship Council (FSC), helps maintain responsible forest management in “non-conflict” areas, ensuring supply chain integrity and compliance with sustainable practices. FSC published details on measures in Ukraine’s “conflict areas” and revised the controlled wood national risk assessment for Ukraine on 4 April 2022 (FSC 2022). For forest management units in potentially hazardous zones, the updated Forest Stewardship Standard proposes excluding contaminated areas from certification (FSC 2024). Certified forestry enterprises are required to implement comprehensive measures, including identifying and mapping contaminated areas, clearly delineating these zones, adapting management plans and monitoring systems, and ensuring public access to non-confidential information. These measures support safety, integrity, and sustainability while addressing the complexities introduced by explosive contamination. Management practices are also adapted to ensure High Conservation Value (HCV) areas remain protected, preventing forest products from contaminated areas from entering FSC-certified supply chains.

While voluntary certification partially compensates for gaps in mandatory policy, regulatory challenges persist. The Ukrainian government has also adjusted logging regulations during wartime, imposing new restrictions but simultaneously removing seasonal logging bans. This change raises sustainability concerns, especially given that clear-cutting accounts for 89% of logging activities (SSSU 2024). Such legislative adjustments could undermine conservation efforts critical to Ukraine’s obligations under the Bern Convention and its alignment with European environmental standards. Analysis of laws, governmental programmes, and their implementation shows that previous policies and strategies did not fully consider the range of ecosystem services, expectations of society to be included in the decision-making, and the impacts of climate change on forest management (Soloviy et al. 2017). While recent policies indicate progress, the evolving regulatory landscape continues to reflect the challenges of balancing immediate needs with long-term sustainability objectives in forest governance.

### Resolution: Pathways for recovery

We identified three strategic pathways for addressing the complex challenges of post-war recovery. They are outlined below and are grounded in the key challenges discussed in the preceding sections. They synthesise recurring themes emerging from our analysis, including increased demand for timber, widespread forest degradation due to fires and UXO, and the urgent need for adaptive governance to build resilience. The pathways are shaped by the pressures and tangible opportunities observed within Ukraine’s forest SES. A detailed summary of how each pathway aligns with specific challenges, analytical dimensions and supporting sources, is provided in Appendix SI 2 and Appendix SI 3.

The first pathway promotes the use of domestically sourced wood and non-wood forest products for post-war reconstruction and green energy, while emphasising the need for sustainable forest management to protect ecosystem services, including in the long term. The second pathway focuses on long-term forest restoration for climate resilience and multi-functionality in delivering ecosystem services to the society with the application of advanced technologies and digital tools to support the process. The third pathway prioritises adaptive and participatory governance and integration of social and socio-ecological innovations to build institutional resilience, ensure transparency, and promote equitable, long-term sustainability. The findings also show that the devastation in eastern Ukraine, including the loss of life of forestry personnel and the destruction of facilities, vehicles, and resources such as natural capital, vital for forest management and use, means that basic recovery (of financing, infrastructure, human- and man-made capital, etc.) must take precedence before the more advanced pathways outlined below in this paper can realistically be pursued.

#### Pathway 1: Increased forest use for sustainable, low-carbon and green recovery

Pathway 1 emphasises the domestic use of Ukraine’s wood to meet urgent needs for building material, primarily for housing reconstruction and new construction of residential houses and infrastructure, with green and low-carbon construction materials such as wood (FSC 2024). It also envisages that timber will be used for ensuring “green” energy supplies, focused on small-scale (residential and municipal) green energy production and high-efficiency wood biomass applications (e.g. Mika and Keeton 2015; Soloviy et al. 2019). The rationale behind an increase in volumes of timber production for wood construction, wooden value-added products and energy was highlighted by, for example, EU New Bauhaus objectives and Ukraine’s Green Deal and Green Policies accordingly (The European Green Deal 2024). However, “*while timber is critical for rebuilding”* (and for small-scale, green energy projects) “*it is essential to ensure that economic recovery efforts do not compromise long-term sustainability*” (Expert 1).

It will be vital that increasing wood supplies meet demands for timber without jeopardising other critical forest ecosystem services such as windbreaks and soil protection, e.g. in eastern Ukraine (where agricultural land dominates), and flood protection in western Ukraine, which features the steep slopes of the Carpathian Mountains, and where clear-cutting has been proven to reduce water regulation, soil protection, habitat provisioning, and climate regulation functions. Thus, meeting the objective of spurring timber production can be achieved only via the widespread adoption of sustainable forest management practices.

We recommend a nationwide *timber sourcing and sustainability feasibility* study (i) to assess the types and quantities of wood needed for post-war reconstruction and increased demand for using wood for energy; (ii) determine the production capacity in different forest regions consistent with harvesting, transportation, processing and manufacturing capabilities; (iii) refine estimates of production capacity after applying spatial filters and sustainability criteria, which would address, at a minimum, protection of high-conservation value forests and biodiversity, hydrologic regulation, and climate resilience; and (iv) provide recommendations for appropriate sourcing of timber locally, regionally, and nationally, consistent with production capacity and sustainability.

The development of value-added forest products will also be essential to meet the demand for wood, create economic resilience and reduce dependency on raw timber extraction. Beyond wood, increased use of non-wood forest products could increase capacities for local food supply generation (through the development of short supply chains and value-added products). Monetisation of non-wood forest products and industries like ecotourism can contribute to economic growth and green job creation, improving the health and wealth of people, and helping to diversify local economies.

However, the first step in implementing Pathway 1 is to address war-related damage, i.e. demining of unexploded ordnance and restoration of damaged forest enterprise and infrastructure. This will require substantial investment, including in modern forestry equipment. Expert 6 stressed that “*Rebuilding the sector will require not just economic investment but also a focus on forest health* (and vitality) *through* (development and use of) modernised (and eco-friendly forest) *management practices*”.

#### Pathway 2: Close-to-nature and close-to-people forestry with technological innovations, digital tools, and new know-how

The “close-to-nature and close-to-people forestry” pathway emphasises long-term ecological restoration and the development of climate-resilient landscapes through close-to-nature forestry, incorporating contemporary digital planning and modelling tools and innovative silvicultural approaches addressing a full range of ecosystem services (Vacik et al. 2020; Rosset 2021; Aszalós et al. 2022; Garcia et al. 2022). It follows principles of an integrated landscape approach, incorporating nature-based and climate-smart solutions (Verkerk et al. 2020; Mathys et al. 2021; Seddon et al. 2021; Cooper and MacFarlane 2023; Wellmann et al. 2023) and highlighting the active participation of local communities in forest management decisions (Waeber et al. 2023).

Given the impacts of the war, such as fire damage and land contamination, efforts should start with demining, improved fire management, nature regeneration, and planting of native species to restore damaged forest ecosystems and ensure forest health. This pathway prioritises restoring biodiversity and enhancing climate resilience. Close-to-nature forestry with multifunctional and sustainable silviculture approaches promotes reforestation with native species and aims to mitigate the risk of fires and pest infestations (Krynytskyi et al. 2017). Expert 4 emphasised that “*restoring forest ecosystems with native species ensures not only biodiversity but also climate adaptation, reducing vulnerability to fires and extreme weather events*”.

Pathway 2 offers slower but more sustainable economic gains by focusing on green job creation and benefits from forest ecosystem services other than timber (e.g. non-timber forest ecosystem services and value-added products). “*Ecotourism, agroforestry, forest playing a role as a shelterbelt for agriculture, and forest-based value-added businesses offer diversified income sources that align with ecological goals*” (Expert 3). These sectors would also provide well-being and resilience for rural communities.

Decentralisation of forest management and empowering local communities is key to this pathway’s success. The Environmental Impact Assessment Law (Law of Ukraine No. 2059-VIII, updated on January 4, 2024) and National Forest Inventory Program (Cabinet of Ministers of Ukraine 2021) provide the necessary regulatory framework to ensure that community-led efforts are aligned with national ecological goals. Expert 5 stated “*Local communities should play a central role in decision-making to ensure forest governance reflects both local needs and national priorities*”.

To enhance forest health and vitality, this pathway focuses on maintaining ecosystem services through proactive monitoring and adaptive management. Preventative measures for fire management and the restoration of war-damaged forests will require continuous investment in forest health technologies. Expert 6 noted that, “*Long-term monitoring and adaptive management will be critical in ensuring that forest ecosystems remain healthy and resilient*”.

Digitalisation and new technologies have significant potential to support sustainable, multifunctional, and nature-based forest ecosystem management (Rosset 2021; Wellmann et al. 2023). Expert 7 admitted that “*digital tools must be fully integrated into the recovery process for forest demining, inventory, and sustainable management, enabling real-time monitoring of forest health and regeneration*”. Tools like terrestrial and airborne laser scanning, remotely sensed imagery, geographic information systems, decision support systems (such as Marteloscope platforms), and simulation modelling, enable integrated, long-term forest planning and monitoring. They aid managers in balancing multiple objectives, as well as anticipating climate vulnerabilities and risks. Knowledge exchange enables sharing of practical experiences, improving evidence-based understanding of forest dynamics and silvicultural techniques, and keeping people well informed about forest health and management practices. Fostering collaboration among forestry stakeholders is crucial for adapting to ongoing challenges, such as climate change.

#### Pathway 3: Anticipatory governance for forest socio-ecological system sustainability with social and socio-ecological innovations

Pathway 3 focuses on refining Ukraine’s forest policies and “*strengthening forest governance, enhancing transparency, and ensuring compliance with sustainable practices to align economic activities with environmental goals*” (Expert 7). It emphasises the building of accountability through regulatory and anti-corruption reforms and leveraging international partnerships to enhance governance capacity. The reforms and their implementation are necessary to prevent overexploitation of natural assets, and promote responsible, integrative and sustainable forest management. Innovative, anticipatory forest governance is deemed to improve transparency, address corruption, and promote decentralised and more deliberative decision-making (e.g. as seen in Muiderman et al. 2020). In this regard, the Program on Anti-Corruption Measures in Forestry (Program on Anti-Corruption Measures in Forestry 2023), which is under implementation, is a critical step in preventing overexploitation of natural capital and steering sustainable governance and management of forests. “*Strong governance prevents deforestation and forest degradation by enforcing sustainable practices and reducing the influence of corrupt actors*” (Expert 3).

Transparency in forest management practices (e.g. through digitalisation) is essential for achieving these goals. Technical or technological innovation, offering digital solutions, such as the Digital Tracking and Certification System introduced in Ukraine in 2022, play a crucial role in enabling real-time monitoring of timber harvesting, and thus increasing the transparency and accountability of forestry operations. “*The use of digital tools to track timber harvesting and sales is essential to maintain transparency and protect forests from over-exploitation*” (Expert 4). Digital wood tracking systems, actively promoted within EU policies like the FLEGT Action Plan (Villanueva et al. 2023), can prevent illegal logging and ensure promotion of transparency in the management and use of timber resources. Continued efforts to decentralise, following the law on decentralisation (2015), could empower local governments to take a more active role in decision-making, creating a more responsive and transparent governance. Expert 5 emphasised “*decentralisation allows local authorities to manage forest resources more effectively, improving accountability and transparency*”. Collaborative forest policy frameworks that involve multi-level engagement of multiple stakeholders contribute to building robust regulatory systems (World Bank 2020; OECD 2022; FAO 2023; Forest Europe 2023; SSSU 2024; World Bank 2024). Capacity building and empowering local stakeholders is a prerequisite for transferring more decision-making powers to local forest authorities and communities. Women are to play an important role in this process (CAS Rebuild Ukraine 2024; Sarkki et al. 2024), including by enabling social and socio-ecological innovations.

Innovative and anticipatory forest governance, built on these collaborative efforts, can strengthen social capital further, steering the diffusion of social innovations (Science Communication Unit 2014; SIMRA 2020; Nijnik et al. 2022). Social and socio-ecological innovation (along with other types of innovation, e.g. technological, digital, financial, governance, institutional) can help address challenges by fostering community participation, the rethinking of governance structures, and by enabling a more integrated decision-making process that incorporates environmental, social, and economic considerations. Social innovation might include the introduction of new rules (formal or informal), governance arrangements, practices, relationships or institutional frameworks. It strives to foster collaborations among actors, strengthening human agency (Lukesch et al. 2020; Weiss et al. 2021).

Social innovation is about the reconfiguration of current social practices and introduction of new ones (Kluvankova et al. 2021), e.g. of social entrepreneurships (Górriz-Mifsud et al. 2019). In favourable institutional settings, it is capable of strengthening sustainable forestry initiatives by aligning them with local needs, promoting biodiversity, and supporting rural livelihoods (Melnykovych et al. 2018; Barlagne et al. 2021; Nijnik et al. 2021; Brnkalakova et al. 2022; Winkel et al. 2022; Živojinović et al. 2023). Social initiatives in Ukrainian forestry have started developing and might indeed help to enhance climate change adaptation and mitigation, promote social justice and inclusion, the creation of jobs and value-added products and services, and alleviate biodiversity losses (Sarkki et al. 2019b; Nijnik et al. 2020, 2021; Brantschen et al. 2023; RURACTIVE 2023; Melnykovych et al. 2024b).

Social, socio-ecological and institutional innovations are the focal points of Pathway 3, as they have the potential to promote civic values and foster transformative changes (Baker and Mehmood 2015; Haxeltine et al. 2017; Kluvánková et al. 2018; Castro-Arce and Vanclay 2020; SIMRA 2020). They are closely linked to the promotion of the anticipatory governance of nature and steering of sustainability through the development of strong human agency (Melnykovych et al. 2018; Dalla Torre et al. 2020). Anticipatory governance is expected to unlock great potential for developing a circular forest economy and bioeconomy, encouraging high technology, and the production of value-added products (Hetemäki and Hurmekoski 2020; Palahí et al. 2020). Anticipatory governance refers to governance processes that use foresight to address uncertain futures, guiding decision-making, and actions in the present (e.g. Boyd et al. 2015). “*Better governance will allow Ukraine’s forests to contribute more sustainably to the economy, developing a full value-added chain rather than relying on raw timber extraction*” (Expert 2). The economic benefits of anticipatory forest governance also include better timber pricing, increased trust in timber certification, and expanded market access as well as innovative forest products. In addressing Ukraine’s future challenges, anticipatory governance is also needed to protect ecosystems from overexploitation during and following the war and to manage possible conflicts among stakeholders due to land-use changes, including those driven by the changing climate which escalate problems associated with wildfires caused by warfare. Expert 4 emphasised that “*the future of Ukraine's forests lies in sustainable landscape management with modern fire-resilient strategies, focusing on both suppression and prevention, particularly in war-affected zones*”.

The principles of ecological economics should provide a foundation for creating effective policies, governance, and management practices for Ukraine’s forest SES recovery (see also (Soloviy et al. 2017). These principles would help forest policy meet societal expectations, with a focus on integrating natural capital considerations into decision-making and stimulating innovations to successfully address government and market failures, currently being observed. Social expectations from governance innovation concern the provision of multiple ecosystem services from forests. This needs to be prioritised, because of the importance of nature for people’s well-being, and as (among other societal benefits) forests play an important role in mental health recovery in post-war times (as seen in Poulsen et al. 2015).

Together, the three recovery pathways outline a strategic vision for Ukraine’s forest socio/ecological system recovery: mobilising forest resources for green reconstruction (Pathway 1), restoring ecosystem resilience through close-to-nature and close-to-people management (Pathway 2), and strengthening governance through anticipatory, transparent and innovation-driven approaches (Pathway 3).

## Discussion

### Forests are cornerstones of resilient post-war recovery

Forests are an integral part of Ukraine’s landscape, cultural heritage, and economy. They provide essential habitat for biodiversity and contribute to the well-being of communities through provisioning of timber and non-timber products. Outdoor recreation, flood control, and climate regulation are other important forest-derived services (Melnykovych et al. 2018). However, challenges in forest SES highlight the need to address and implement rapid forest recovery solutions (Lugano Declaration 2022; Forest Europe 2023; FAO 2023). These solutions must be implemented taking into consideration lessons learnt from other post-war countries (da Costa et al. 2023), emphasising resilience in SES, as seen in both agricultural (Nehrey and Finger 2024) and forest sectors (Zibtsev et al. 2024a).

Anticipatory forest governance, which incorporates foresight in decision-making, could empower Ukraine to respond to climate change and its economic, and socio-environmental impacts (Boyd et al. 2015; Muiderman et al. 2022). It could enable Ukrainian forest governance and management to balance ecological components of sustainability with its economic and social imperatives, supporting long-term resilience of SES. Regulated commercial forest management generates needed short-term economic value, such as providing timber as a renewable resource for wood-based construction, emphasised by the European Commission (European Commission 2022) in new EU Bauhaus initiative. Close-to-nature, multifunctional forestry ensures these activities are balanced with ecological sustainability, resulting in an integrated approach that would advance both socio-economic and environmental objectives in Ukraine’s post-war recovery (as outlined in Ministry of Economy of Ukraine 2024; Ministry of Environmental Protection 2024; Plan for the Recovery of Ukraine 2024).

War-related pressures on Ukraine’s SES and economy include not only the destruction and contamination of forests (Pereira et al. 2022), particularly in the eastern and central regions, but also a rising demand for timber, especially for reconstruction (Milakovsky 2024), which, combined with increasing needs for energy from forest biomass, further exacerbates the situation (Soloviy et al. 2019; Melnykovych et al. 2020). Moreover, in Ukraine, many of the destroyed forests served as shelterbelts or windbreaks, protecting huge agricultural fields, thus supporting food provision and security (Yukhnovskyi et al. 2021; Yaroshchuk et al. 2023). Ukraine’s forests previously made substantial and cost-efficient contributions to climate change mitigation (Nijnik 2010). However, today, they have become a source of carbon emissions due to the increased number of forest fires (Zibtsev et al. 2023b).

Finally, conserving, restoring, and sustainably managing Ukraine's forests are crucial objectives that align with many EU and international policy frameworks, although Ukraine’s recovery trajectory will also be shaped by its own priorities, sociopolitical context, and local institutional capacities (United Nations 2015; World Bank 2020; UNECE and FAO 2021; UNEP and FAO 2021; European Commission 2023; Achasova and Achasov 2024).

### Forests offer diverse values beyond timber extraction

Post-war conditions often require rapid recovery to stabilise human health and well-being. Natural resource extraction through large-scale industrial logging can drive economic growth and support recovery. In Finland, intensive forestry practices after World War II contributed significantly to recovery, with forests becoming a foundational asset for growth (Metsähallitus 2024). Today, many forest ecosystems have declined, biodiversity has been lost, and carbon sequestration in forests has decreased (Määttänen et al. 2023). Nevertheless, industrial forestry was and still is important for the Finnish economy.

Ukraine is in a different situation. Similar to the recovery needs of its agricultural sector, Ukraine’s forestry can leverage integrated, resilient practices to support sustainable growth, balancing immediate economic requirements with long-term sustainability objectives, for meeting important ecosystem health and vitality needs (Nehrey and Finger 2024). Beyond timber production, Ukraine’s forests offer other economic opportunities (e.g. carbon markets). While industrial timber production and carbon forestry can provide an important source of jobs, income, and economic growth, it is not the only way for forests to contribute to long-term sustainability, particularly under the climatic and other conditions of Ukraine where forests provide important regulatory and protective functions. Thus, combining timber production with multi-purpose close-to-nature forestry can provide a variety of societal gains, especially at the local level. These include protecting high-value agricultural lands (e.g. through agroforestry as per Elbakidze et al. 2024) and enhancing cultural and traditional values provided by wooded landscapes (Martino et al. 2024).

Advancing forest research and education is central in this regard. Strategic forest science efforts build an evidence base for guiding recovery strategies, while innovations in education and academic exchange equip new forest professionals to meet Ukraine's challenges (Zibtsev et al. 2024a, 2024b).

Important considerations need to be made to address the current market imbalance, characterised by the shift towards fuel wood as a dominant segment within Ukraine’s timber market which reflects complex underlying dynamics that go beyond simple supply and demand. While domestic market forces and local economic pressures contribute to this trend, broader geopolitical factors likely play a role (World Bank et al. 2024). With Ukraine aiming for EU integration, there is an incentive to align with Europe’s movement away from fossil fuels and non-renewable energy. This transition has created increased demand for energy wood, potentially putting pressure on Ukraine’s forest resources, particularly the Carpathian forests, which are unique for their biodiversity and non-market values (Hamor 2023) as well as being economically accessible and conveniently located for export. The situation and results of our analysis suggest that wood extraction in Ukraine may be influenced by external market demands and geopolitical motivations, adding a political layer to what might otherwise seem a domestic issue. Although this analysis focuses on Ukraine’s internal market conditions, the broader context highlights the need for a balanced approach that considers environmental sustainability and the long-term impact of increased forest utilisation for energy.

Often a clear link between socio-economic resilience and nature restoration, particularly for social groups or communities that are dependent on forests for their livelihoods, needs to be considered in recovery practices (Adams et al. 2016; Padovezi et al. 2022). The Ukrainian case demonstrates that investments and aid from the EU, based on green practices, create an additional link between ecological and socio-economic resilience, extending beyond the local level. The alignment of Ukrainian forest management with EU biodiversity and climate goals can increase access to EU funding, like green and climate finance, which can be important sources of financial aid and investments in the future. Furthermore, the lure of short-term economic gains from industrial timber production and using wood for energy could compromise both ecological sustainability and economic opportunities in the long term. Maintaining the carbon sequestration and storage capacity of Ukraine’s forests (Nijnik 2010), including for helping to offset emissions from post-war reconstruction, is important within an integrated forest management framework (Kruhlov et al. 2018; Verkerk et al. 2020).

### Relying on a single recovery pathway risks long-term vulnerability

Integrating multiple recovery pathways and anticipatory governance creates synergies. Recent research emphasises the pivotal role of institutional and governance arrangements, alongside stakeholder engagement and social innovations in fostering resilience within forest SES (Melnykovych et al. 2018; Sarkki et al. 2019a; Ludvig et al. 2020; Nijnik et al. 2021, 2022; Weiss et al. 2021; Winkel et al. 2022; Živojinović et al. 2023). Socio-ecological innovations can emerge by reconfiguring traditional governance practices and integrating societal expectations into policies and institutions (Melnykovych et al. 2018). This approach can help tackle the complex challenges of post-war forest recovery in times of crises, like in Ukraine (Secco et al. 2017; Weiss et al. 2021; Winkel et al. 2022; Živojinović et al. 2023).

Self-reinforcing feedback mechanisms are vital for building resilience. They help SES maintain their core functions or adapt to disruptions in ways that preserve their integrity over time (Olsson et al. 2004; Chaffin and Gunderson 2016). Resilience comes in different forms. Sometimes it is about bouncing back—recovering to a prior state. But other times, it is about bouncing forward—adapting, learning, and transforming into something “better,” more sustainable (Gunderson and Holling 2002; Walker et al. 2006). For Ukraine, the latter is key. Recovery here is not just about forests; it is about securing socio-economic well-being and creating space for people to shape the future through inclusive decision-making.

This is where anticipatory governance comes in. At its core, it is about acting today to navigate the uncertainties of tomorrow (Muiderman et al. 2020). It does not rely on rigid plans but explores multiple possibilities, aligning present-day actions with long-term goals. In Ukraine’s case, this means grappling with a range of challenges—physical infrastructure, the social fabric of rural communities, and human lives shattered by the war; climate change; increasing demand for timber and energy under “green” policies; market shifts; and shifting societal expectations for forests. The way forward demands evidence-based strategies, collaborative policymaking, and active engagement with stakeholders at every level.

Anticipatory governance (e.g. improving future-preparedness to navigate uncertainties) strengthens resilience (Boyd et al. 2015; Muiderman et al. 2020; Spitz 2024), aligning Ukrainian forest policy with EU policies (Forest Europe 2023; FAO 2023). Here, we observe that the three identified pathways—“increased forest use for sustainable, low-carbon and green rebuilding for housing reconstruction, and for small-scale, green energy production”, “close-to-nature and close-to-people forestry with technological innovations, digital tools, and new know-how”, and “Anticipatory governance for forest socio-ecological system sustainability with social and socio-ecological innovations”—have linkages to one another, and when they occur simultaneously it will reinforce resilient recovery. The three pathways, when combined, have the potential to create reinforcing feedback loops that enhance the resilience and multi-functionality of forests (similar to Katila et al. 2024).

Governance reforms provide a regulatory foundation for transparency (FLEG 2010) and for the fostering of public–private partnerships that align with community needs and promote social justice in resource distribution (Loveridge et al. 2023). These reforms, when deliberately embracing social innovation, can promote equitable resource distribution and encourage community involvement in forest management, ensuring sustainable use of resources (Kluvánková et al. 2018). Capacity building, along with the development and sharing of knowledge (Kluvánková et al. 2018), including new technologies and approaches, is likely a key driver in shaping innovative solutions (Špaček et al. 2022; CAS Rebuild Ukraine 2024; Melnykovych et al. 2024b). Sustainable and inclusive landscape governance dialogues, which adopt a holistic perspective by considering the intricate interconnections between the environment, economy, and society, should be firmly rooted in principles of inclusiveness, transparency, and collaboration (Garcia et al. 2022; Waeber et al. 2023).

Institutional reforms will play a vital role in boosting the economy, strengthening public–private partnerships in forest use, and creating robust forest management rules (Vanegas-Cubillos et al. 2022)—such as measures to prevent the re-emergence of pre-war issues like illegal logging or risks of non-transparent governance (Nijnik et al. 2021). Social innovations and the development of “green” jobs, as well as the promotion of new business opportunities, are integral to fostering resilience (SIMRA 2020). Attention to forest as a provider of opportunities for mental health recovery and community well-being is also essential, as these factors contribute to social cohesion and the overall sustainability of recovery efforts (Poulsen et al. 2015; United Nations 2015; UNEP and FAO 2021).

There is a need to continue advancing a multidisciplinary strategy for Ukraine’s recovery, integrating sustainable forest management (of which close-to-nature forestry can be an important component) and governance reform. This approach emphasises diverse pathways and adaptable policies to address environmental, social, and economic challenges, building resilience by supporting ecosystem services, biodiversity, and economic growth (Chan et al. 2016; Katila et al. 2024). It is crucial to recognise the costs and trade-offs involved, reinforcing multiple strategies rather than reliance on a single pathway. International commitments and technological innovations should be leveraged to guide recovery. However, transformative change occurs when anticipatory governance is implemented (Muiderman et al. 2022), nature is recognised as central to our economy (Palahí et al. 2020), the diverse values of nature are incorporated into decision-making (Gómez-Baggethun et al. 2013; Sarkki et al. 2019b), and the values, beliefs, knowledge (Palomo et al. 2024), and agency of all stakeholders are recognised and integrated (Sarkki et al. 2019b; Garcia et al. 2020).

## Concluding remarks

In response to Ukraine’s urgent post-war recovery needs, efforts must balance immediate timber demands with sustainable approaches for landscape recovery, and close-to-nature forestry practices that align with EU climate and biodiversity goals. Emphasising adaptive strategies, we suggest that recovery should integrate strong participation and enable social innovations. Ukraine’s forest SES recovery must envisage forest (circular) bioeconomy, creating green jobs, supporting multi-purpose and climate-smart forestry, and advancing policies that promote forest SES resilience. To meet future challenges, anticipatory forest governance will be crucial, incorporating foresight to address the impacts of climate change, socio-environmental pressures, and the demands of post-war reconstruction. Forest science and education are central to ensuring this outcome, helping to elaborate robust evidence-based strategies by integrating digital solutions, the latest know-how and technologies and preparing the next generation of professionals for sustainable forest management, including via better integration of women in the forest sector and overall recovery process. Engaging in international research and policy networks, as identified in the recent IUFRO Forum, can support Ukraine’s forestry integration into EU and international forest policy and governance standards, promote science-based recovery strategies, and enable international support.

Appropriate integration of building blocks from the different recovery pathways identified in this paper, and the enablement of green policies, social innovations and anticipatory governance, will create synergies and strengthen the recovery of forest SES especially under ongoing geopolitical pressures, climate change challenges and market fluctuations. Active involvement of diverse stakeholders in the recovery process is a prerequisite for achieving a sustainable, resilient and multifunctional forest SES in post-war Ukraine.

## Supplementary Information

Below is the link to the electronic supplementary material.

Supplementary file1 (DOCX 376 KB)
