# Supplementary material for: Pathways for Ukraine’s post-war nature recovery: Focus on forest socio-ecological systems
Source: Ambio. 2025 Oct 23;55(4):817–43. doi: 10.1007/s13280-025-02263-0 (PMC12961059; doi:10.1007/s13280-025-02263-0)
Supplement: Supplementary file 1 — Supplementary file1 (DOCX 376 KB) [file 13280_2025_2263_MOESM1_ESM.pdf]

Supplementary Information

This Supplementary Information has not been peer reviewed

Title: Methodological procedure for identifying recovery pathways in the manuscript "Pathways for Ukraine's Post-War Nature Recovery: Focus on Forest Socio-Ecological Systems"

Appendix SI 1: Questionnaire applied to interview forestry experts in Ukraine

Dear Experts,

We at the School of Agriculture, Forestry and Food Sciences HAFL at the Bern University of Applied Sciences BFH, are conducting a questionnaire to understand major challenges that forest socio-ecological systems in Ukraine have been facing in **2004-2024**. We seek your insights to understand the complexities of Ukraine's forestry during this period and in post-war time.

Your expertise will contribute to improving of our understanding of the state of the affairs and identification of potential pathways for recovery.

This questionnaire is built on the five-steps (sections) analytical framework that includes defining the nature of the crisis (What?), assessing impacts (How Much?), identifying root causes (Why?), evaluating actions (Who?), and developing recommendations (Where?).

Please kindly answer the following questions:

Section 1: Understanding the Challenges associated with Ukraine's forests and forestry

1. Before the war in Ukraine, in years 2004-2024, what, in your view, were the *challenges faced by Ukraine's forestry sector*? (list please and describe with some examples and links/ references)

1.1. How did these challenges affect the *forest ecosystems in Ukraine before the war*?

1.2. How did these challenges affect the *forest economy*?

1.3. What were the influences of these challenges on *forest-dependent communities and society prior to the war*?

1.4. How did these challenges influence *forest policies and governance* in Ukraine before the full-scale war?

2. What are the five biggest impacts of the war on the forest sector? (list, prioritise and describe with some examples)

3. How does the war influence the forestsector? (can you please provide some examples? Share references or links to the information?)

3.1. What is the influence of the war on Ukraine's forest ecosystems?

3.2. What is the influence on forest economy

3.3. What is the influence of the war on forest-dependent communities and society?

3.4. What is the influence of the war on Ukraine's forest policies and governance?

4. What is your perspective on the ongoing forestry reforms, and how do you evaluate their impact?

4.1. Which specific aspects of the forestry reforms do you view as positive, and why?

4.2. Conversely, which aspects of the forestry reforms do you consider negative, and why?

Section 2: Key Actors and Interactions

5. Can you list the *specific institutions or organisations* that are important players in addressing the challenges you have identified? (see Questions 1 and 3)

Section 3: Recommendations and Opportunities for the Future

6. Based on your understanding of the challenges, what *recommendations or strategies* would you propose to prepare the forest sector for post-war times in Ukraine?

7. What do you think are the *most needed changes* in the forest sector to address the challenges?

8. How do you envision the *forest sector contribution to the post-war recovery* of Ukraine?

Thank you for the interview. Your interview will be analysed and kept confidential.

International Forest Governance Team

School of Agriculture, Forestry and Food Sciences HAFL

Bern University of Applied Sciences BFH

Appendix SI 2: Coding expert interviews

This appendix documents the analytical process and highlights how expert input contributed to identifying recovery priorities. Expert interview responses (n = 7) were analysed using **inductive thematic coding** in QDA Miner Lite. Individual statements were coded based on recurring ideas, with codes grouped into broader thematic categories through iterative review. Table SI 2.1 summarises the resulting themes, their frequency across interviews, and their

alignment with the recovery pathways described in Section 3.4. Table SI 2.2 presents selected verbatim quotes that reflect how expert statements were assigned to each theme.

Table SI 2.1. Thematic codes and their frequency across expert interviews. Each theme was derived inductively from expert responses using QDA Miner Lite. Codes were grouped into broader thematic categories aligned with one of the three recovery pathways presented in Section 3.4 of the manuscript.

**Code**	**Frequency**	**Aligned pathway**
Governance and Transparency	12	Governance Reform
Corruption	10	Governance Reform
Institutional Weaknesses	8	Governance Reform
Community Engagement	8	Governance Reform
Economic Recovery	6	Economic Recovery
Timber Production	5	Economic Recovery
Sustainable Forest Management	9	Ecological Restoration
Biodiversity Restoration	7	Ecological Restoration
Fire and Forest Health	5	Ecological Restoration
Digital Tools and Monitoring	4	Cross-cutting (Gov + Eco)

Table SI 2.2 Quotes by theme. Selected interview excerpts illustrate how expert input supports the thematic categories used in the analysis. Quotes are presented unedited or translated ad verbatim.

**Theme**	**Verbatim quote**	**Expert**
Governance/ Transparency	"Digital tools must be fully integrated into the recovery process... enabling real-time monitoring of forest health and regeneration."	Expert 7
Governance/ Transparency	"Lack of adequate policy and legislation to regulate forest management, including ecological."	Expert 6
Governance/ Transparency	"Відсутність стабільності у законодавчій базі ускладнювала планування діяльності..." >> "The lack of stability in the legislative framework complicated activity planning..."	Expert 7

Governance/ Transparency	"Transparency (not formal, real one) will decrease the risks of corruption, improve accountability, and promote fair forest governance."	Expert 2
Corruption	"Strong governance prevents deforestation and forest degradation by enforcing sustainable practices and reducing the influence of corrupt actors."	Expert 3
Institutional Weaknesses	"These reforms primarily emphasised increasing economic efficiency through too centralised management functions..."	Expert 3
Community Engagement	"Local communities should play a central role in decision-making to ensure forest governance reflects both local needs and national priorities."	Expert 5
Community Engagement	"Lack to almost absence of participatory planning of forest management... absence of trust towards foresters and their work due to negative past..."	Expert 6
Community Engagement	"Важливим є розвиток так званих «лісових терапій», еколого-терапевтичних прогулянок... сприяє як покращенню ментального стану, так і неформальній екологічній освіті..." >> "The development of so-called 'forest therapies'... supports mental well-being and informal environmental education..."	Expert 7
Timber Production	"триває значне зростання попиту на деревину" → "There is a continued significant increase in demand for timber."	Expert 4
Economic Recovery	"Timber resources for rebuilding initiatives."	Expert 6
Sustainable Forest Management	"It is essential to ensure that economic recovery efforts do not compromise long-term sustainability."	Expert 1
Sustainable Forest Management	"Education of forestry practitioners regarding close-to-nature silviculture practices and approaches."	Expert 6
Sustainable Forest Management	"Практика наближеного до природи лісівництва... є те що потрібне буде лісовій галузі..." >> "The practice of close-to-nature forestry... is what will be needed by the forestry sector..."	Expert 7
Biodiversity Restoration	"Restoring forest ecosystems with native species ensures not only biodiversity but also climate adaptation..."	Expert 4
Biodiversity Restoration	"Роботу над збереженням біологічного різноманіття в лісах та розвиток природно-заповідного фонду слід вважати позитивним..." >> "The work on conserving biodiversity in forests and developing the nature reserve fund should be considered positive..."	Expert 7

Fire and Forest Health	"Fire ignition and spread are mainly caused by continuous shelling, as well as the absence of forest fire management and suppression systems."	Expert 1
Fire and Forest Health	"Increase of forest fires due to rocket shelling."	Expert 6
Fire and Forest Health	"Forest ecosystems were significantly affected by fire and destruction, including forest cover loss due to military actions."	Expert 4
Digital Tools and Monitoring	"The use of digital tools to track timber harvesting and sales is essential to maintain transparency and protect forests from over-exploitation."	Expert 4

Appendix SI 3. Synthesis of information to identify the three recovery pathways

The following Table SI 3.1. synthesises how each of the three recovery pathways (outlined in Section 3.4) is grounded in the analytical framework and empirical findings presented earlier in the manuscript. The pathways are not conceptual abstractions but were developed in response to observed pressures and institutional challenges affecting Ukraine's forest socio-ecological system. They build on the integrated analysis structured around the three key dimensions used in this study: Nature of the crisis, scope of the crisis, and resolution of the crisis (cf. Section 2, Table 1 in the manuscript). These three dimensions guided the identification of pre-war vulnerabilities, war-related disruptions, and areas requiring policy or institutional response.

The organisation of insights into three distinct recovery pathways follows the structure proposed by Beland Lindahl et al. (2015, 2017), who emphasise that sustainability pathways should emerge from context-specific crisis framings and reflect the interplay of social, ecological, and institutional domains. Accordingly, the three pathways focus on: socio-economic recovery (Pathway 1), ecological restoration (Pathway 2), and anticipatory governance and innovation (Pathway 3).

The three pathways are primarily grounded in published literature and policy documents, with expert interviews used to refine, support, and deepen the analysis. Expert responses (n = 7) were analysed using inductive thematic coding with QDA Miner Lite. The coding process and associated themes are presented in Appendix SI 2, which includes a summary of the codes (Table SI 2.1) and selected verbatim interview excerpts grouped by theme (Table SI 2.2). These themes serve as the basis for linking expert insights to each recovery pathway.

The table below references specific sections of the analysis (Sections 3.2 and 3.3 in the manuscript) and supporting literature to clarify how each challenge was identified and substantiated. In doing so, it completes the analytical arc of the paper by linking the diagnostic framework and empirical material to the strategic recovery pathways presented in Section 3.4.

Table SI 3.1. Synthesis on how each of the three recovery pathways is grounded in the analytical framework and empirical findings presented in the manuscript

**Pathway**	**Challenges addressed**	**Sections of analysis**	**Codes (from Table SI 2.1, Appendix BSI 2**	**Expert input / supporting sources**
**Pathway 1:** Recovery using forest products and wood biomass as the main resources for housing reconstruction and small scale, green energy production	Rising timber demand; need for fuelwood and bioenergy; reconstruction pressures; limited equipment and infrastructure	3.2.2 (Economy), 3.3.2 (Forest Use)	Timber Production, Economic Recovery	Expert 1, Expert 4, Expert 6; SFRA 2024a; FAO 2023; UNEP 2023; Zibtsev et al. 2023a
**Pathway 2:** Close-to-nature and close-to-people forestry with technological innovations, digital tools, and new know-how	Forest degradation; biodiversity loss; fire risk; need for native species; ecological complexity loss; low capacity for restoration	3.2.2 (Environment), 3.3.1 (Forest Ecosystem)	Fire and Forest Health, Biodiversity Restoration, Sustainable Forest Management	Expert 2, Expert 3, Expert 4; Zibtsev et al. 2023a; Forest Europe 2023; WWF 2023; FAO 2023

**Pathway 3:** Participatory governance for forest socio-ecological system sustainability with social and socio-ecological innovations	Centralisation of decision-making; lack of transparency; corruption risks; limited local participation; unclear mandates and reform gaps	3.2.1 (Governance), 3.3.2 (Governance and Society)	Governance and Transparency, Corruption, Institutional Weaknesses, Community Engagement	Expert 1, Expert 2, Expert 3, Expert 5, Expert 7; Nijnik et al. 2021; FAO 2023; OECD 2022; World Bank 2024

The first pathway centres on using forest resources to drive immediate economic recovery. The second emphasises sustainable, long-term forest restoration, focusing on biodiversity and climate resilience. The third pathway prioritises decentralised recovery strategies with decision-making authority vested in local governments.
